# Strategies to identify long noncoding RNAs involved in gene regulation

**DOI:** 10.1186/2045-3701-2-37

**Published:** 2012-11-06

**Authors:** Catherine Lee, Nobuaki Kikyo

**Affiliations:** 1 Stem Cell Institute, Department of Genetics, Cell Biology and Development, University of Minnesota, Room 2-216, MTRF, 2001 6th St. SE, Minneapolis, MN, 55455, USA

**Keywords:** Immunoprecipitation, ENCODE, Long noncoding RNA, Microarray, RNA-seq, Tiling array

## Abstract

Long noncoding RNAs (lncRNAs) have been detected in nearly every cell type and found to be fundamentally involved in many biological processes. The characterization of lncRNAs has immense potential to advance our comprehensive understanding of cellular processes and gene regulation, along with implications for the treatment of human disease. The recent ENCODE (Encyclopedia of DNA Elements) study reported 9,640 lncRNA loci in the human genome, which corresponds to around half the number of protein-coding genes. Because of this sheer number and their functional diversity, it is crucial to identify a pool of potentially relevant lncRNAs early on in a given study. In this review, we evaluate the methods for isolating lncRNAs by immunoprecipitation and review the advantages, disadvantages, and applications of three widely used approaches – microarray, tiling array, and RNA-seq – for identifying lncRNAs involved in gene regulation. We also look at ways in which data from publicly available databases such as ENCODE can support the study of lncRNAs.

## Long noncoding RNAs

**Figure 1:**
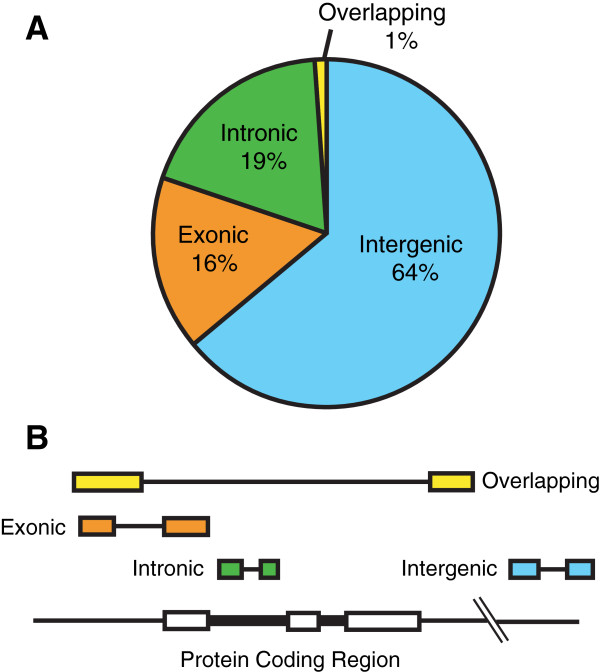
**Overview of lncRNA populations depending on the locations on the genome.** LncRNAs can be categorized into subgroups of intergenic, exonic, intronic, and overlapping according to where they are found relative to nearby protein-coding genes. (**A**) Proportion of lncRNA subgroups [3]. (**B**) Location of each type of lncRNA.

Long noncoding RNA (lncRNA) is operationally defined as RNA longer than 200 bases that does not encode mRNA, rRNA or tRNA [1,2]. Although several lncRNAs have been sporadically identified and characterized in the past 20 years, genome-wide identification of lncRNAs has only recently become possible with the advent of high-throughput sequencing technologies of cDNA (RNA-seq). Evidence that this field is gaining momentum can be seen in the most recent report of the ENCODE (Encyclopedia of DNA Elements) project published in September 2012, which described 9,640 lncRNA loci in comparison to 20,687 protein-coding genes in 15 human cell lines [3-5]. This ratio of lncRNAs and protein-coding genes underscores the potential magnitude and diversity of the biological effects mediated by lncRNAs. Indeed, despite the fact that only about 100 lncRNAs have been functionally characterized to date [4], it has become clear that lncRNAs are involved in almost every aspect of cellular and molecular biology. LncRNAs control cell differentiation, development, cancer progression, and cell metabolism, among other cell functions. At the gene expression level, lncRNAs regulate all processes of RNA metabolism including chromatin modification, transcription, splicing, RNA transport, and translation. LncRNAs themselves are transcribed from intergenic regions, exons, introns, and their overlapping regions (Figure 1A and 1B). At the mechanistic level, lncRNAs serve as “scaffolds” providing platforms to assemble RNA-protein complexes, “guides” to recruit RNAprotein complexes to target genes, and “decoys” by binding to and sequestering regulatory proteins away from their target DNA sequences [1,2]. Given the recent appreciation for the biological importance of lncRNAs, it is now clear that, regardless of the research project or field, one needs to ask whether lncRNAs are essential mechanistic components of the biological process under consideration. The first step to addressing this question is to identify lncRNAs that are potentially relevant to the research field. The current review article provides an overview of four widely used approaches to identify lncRNAs involved in gene regulation – immunoprecipitation of RNA and chromatin, microarray, tiling array, and RNA-seq – and discusses the advantages and disadvantages of each approach. These approaches, which are not mutually exclusive and are often combined in a single study, have been successfully used to identify lncRNAs (Table 1). The focus of this review is gene regulation, which has been the main area of functional studies of lncRNAs; however, lncRNAs are also involved in the organization of cellular structure and subcellular organelles. For further information on these and other aspects of lncRNA biology, readers are referred to the following recent reviews: [1,2,6-11]. 

**Table 1:** Examples of lncRNAs discovered with various approaches described in the text

**lncRNA name**	**Types of RNA**	lncRNA identification methods*	**Reference**
ANRIL	RNA-ChIP (UV cross-linking) and CLIP	RT-PCR	[12]
RepA	RIP (no cross-linking)	Northern blotting	[13]
TRE-1, -2 and -3	RNA-ChIP (cross-linked with formaldehyde)	RT-PCR	[14]
Xist	RIP (no cross-linking)	RT-PCR	[13]
LincRNA-EPS	Polyadenylated RNA	Microarrays of 10,802 lncRNAs and RNA-seq	[15]
lincRNA-SFMBT2, lincRNA-RoR, and lincRNA-VLDLR	Total RNA	Microarrays of 900 lincRNAs	[16]
lncRNA_ES1, ES2 and ES3	Total RNA	Microarrays of 6,671 lncRNAs	[17]
ncRNA-a1-7	Polyadenylated RNA	Microarrays of 3,019 lncRNAs	[18]
HOTAIR	Polyadenylated RNA	Tiling arrays of 39 *HOX* genes	[19]
	RIP (no cross-linking)	Validation with RT-PCR	
HOTAIRM1	Polyadenylated RNA	Tiling arrays of *HOXA* genes	[20]
lincRNA-p21	Polyadenylated RNA	Tiling arrays of 400 lincRNAs	[21]
Mistral	RNA-ChIP (no cross-linking)	Tiling arrays of chromosomes 6, 8 and 16	[22]
300 lincRNAs	RIP (no cross-linking)	Tiling arrays of 900 lincRNAs	[23]
1,600 lincRNAs	Polyadenylated RNA	Tiling arrays of 350 K4-K36 domains	[24]
ANCR	Polyadenylated RNA	RNA-seq	[25]
BANCR	Polyadenylated RNA	RNA-seq	[26]
5 lncRNAs	CLIP	RNA-seq	[27]
216 lincRNAs	RIP (no cross-linking)	RNA-seq	[28]
	RIP (UV cross-linking)	Validation with RT-PCR	
>8,000 lncRNAs	Polyadenylated RNA	Analysis of existing RNA-seq data	[29]

* LncRNAs are categorized based on the methods of their discovery: gene-specific approaches (RT-PCR and Northern blotting), microarrays, tiling arrays and RNA-seq.

## Collection of lncRNAs by immunoprecipitation

The first challenge in studying lncRNAs is how to collect RNA pools that potentially contain lncRNAs of interest. One can prepare RNA pools by simply isolating total RNA from cells or tissues in an unbiased manner; however, immunoprecipitation-based approaches are also commonly used to enrich lncRNAs associated with specific proteins. RNA immunoprecipitation (RIP) can be performed with or without cross-linking whole cellular components before making cell extracts. Without cross-linking, one can isolate lncRNA complexes already existing in soluble form and those that can be readily dissociated from chromatin. Zhao et al. used RIP of polycomb repressive complex 2 (PRC2), a key regulator of epigenetic silencing, without cross-linking and co-immunoprecipitated the lncRNA Xist, which was amplified by RT-PCR [13]. Using the same procedure, they discovered co-immunoprecipitation of the novel lncRNA RepA, which is transcribed within the Xist locus [13]. To identify unknown lncRNAs by RIP, the co-immunoprecipitated RNA pool can be applied to microarray analyses or RNA-seq, as described later [19,23,28]. If one needs to exclude the possibility of indirect interactions between lncRNAs and proteins through their binding to neighboring DNA sequences, the immunoprecipitated materials can be treated with RNase H (digests RNA in RNA-DNA hybrids) and DNase I prior to elution of co-immunoprecipated molecules. As a control, treatment with RNase A, RNase I (both digest single-stranded RNA), and/or RNase V1 (double-stranded RNA) should abolish the co-immunoprecipitation [22,28].

There are several RIP techniques that employ cross-linking. RIP is sometimes performed after ultraviolet (UV) irradiation of cells, which cross-links RNA and protein (pyrimidines and Cys, Lys, Phe, Trp, and Tyr) but not protein and protein [30]. This unique feature allows for the recovery of lncRNAs that directly interact with the immunoprecipitated protein. Taking advantage of this high specificity, UV cross-linking is used to identify the domains within an RNA molecule responsible for the interaction with the protein partner. For instance, Zhao et al. irradiated cells with 254 nm UV prior to making cell extracts and immunoprecipitated PRC2 to identify directly associated lncRNAs [28].

A related variation is called CLIP (cross-linking and immunoprecipitation), which was designed to isolate a protein-interacting domain within a given RNA molecule after using a stringent wash to reduce non-specific binding [30]. In a typical CLIP experiment, extracts are made from cells after UV-irradiation and treated with RNase to retain only the RNA region protected by the interacting protein. The partially digested RNA pool is then tagged with a 3’ linker and also radio-labeled. After purification of the protein with immunoprecipitation, SDS gel electrophoresis, autoradiography, and band excision, the bound protein is removed by proteinase K treatment. The exposed RNA is tagged with a 5’ linker and PCR-amplified to identify the sequence. CLIP was successfully used to immunoprecipitate five intronic lncRNAs directly associated with the PRC2 complex [27].

Cross-linking with UV or formaldehyde followed by fragmentation of chromatin is used to immunoprecipitate RNA-chromatin complexes (RNA-chromatin immunoprecipitation or RNA-ChIP) [12,14,22]. While this approach potentially detects false-positive interactions between RNA and protein through DNA as described above, it can be useful to identify lncRNAs that bind to specifically modified histones which require chromatin fragmentation for extraction.

For any of these immunoprecipitation-based approaches, specificity and affinity of the antibodies are decisive factors for the success or failure of the projects. While the specificity of the antibodies is commonly verified by detecting only one band in western blotting, the antibodies may react with other proteins when detergents are used at a low concentration during immunoprecipitation. One solution to address the specificity issue is to use multiple antibodies against the same protein and select reproducibly co-precipitated lncRNAs for further study. Similarly, immunoprecipitation of several different subunits within a single protein complex is also an option to identify lncRNAs that are likely to be genuinely interacting with the complex.

## Identification of lncRNAs with microarrays

Microarray-based approaches and RNA-seq are two of the most commonly used genome-wide screening methods to identify lncRNAs that might be relevant to a specific biological question. Although a tiling array should be included in the microarray section by definition, it will be discussed separately in the next section as it is frequently used for different purposes. Because traditional microarrays can only detect the presence or absence of known lncRNAs in an RNA pool, they are inherently incapable of identifying novel lncRNAs. Inability of distinguishing different splicing variants is another disadvantage of microarrays unless probes encompassing exon-exon junctions are present on the chip. However, given the cost and complexity of the analysis of RNA-seq data, microarray remains the first choice in many applications [15-18]. In particular, since the identification of 9,640 lncRNA loci as part of the ENCODE project, the comprehensiveness of microarrays for human lncRNAs has been drastically improved.

Data generation with microarrays is relatively easy compared to the subsequent step of selecting potentially important lncRNAs from the positive probes on the arrays because the majority of identified lncRNAs remain uncharacterized. Here, the work by Loewer et al. serves as an exemplary case study of how to narrow down lncRNA candidates relevant to one’s interest, in this case, association with pluripotency [16]. Loewer and colleagues designed a microarray containing 900 long intergenic noncoding RNAs (lincRNAs) and hybridized them with total RNA prepared from several different cell lines to identify induced pluripotent stem cell-specific lincRNAs. In their case, the selection criteria included the genomic location (close to the binding sites of pluripotency transcription factors), nearby presence of epigenetic markers for active transcription, behavior of the lincRNA level during differentiation, and consequence of up- and downregulation in terms of the maintenance or acquisition of pluripotency. Similar concepts can be widely applied to selecting lncRNAs in other contexts.

## Identification of lncRNAs with tiling arrays

Unlike traditional microarrays, DNA tiling arrays contain oligonucleotide probes encompassing an entire length of a defined DNA region. Resolution of the hybridized genomic DNA sequence can be adjusted by changing the length of the overlapping sequences between two neighboring probes. A major advantage of using tiling arrays is their capacity to identify novel lncRNAs in a selected DNA region without prior knowledge of their precise locations within the region. The DNA region can be defined by the residing genes of interest. For instance, Rinn et al. focused on lncRNAs expressed in the region of the human *HOX* genes and compared skin fibroblasts isolated from different anatomical regions of the body [19]. They printed 400,000 probes of 50 bases in length with each probe overlapping the next one by 45 bases to cover all four human *HOX* gene clusters. This configuration allowed for the identification of hybridized DNA sequences at 5-base resolution. Polyadenylated RNAs prepared from fibroblasts were then hybridized to the tiling arrays, resulting in the discovery of the lncRNA HOTAIR transcribed from an intergenic region within the *HOXC* cluster. A similar *HOX* tiling array was used to identify lncRNAs specifically expressed in metastatic breast carcinoma [31]. The lncRNA HOTAIRM1 was discovered in the intergenic region between the *HOXA1* and *HOXA2* genes with commercially available tiling arrays covering the human *HOXA* gene cluster [20].

The DNA regions of interest can also be determined by the unique epigenetic features of the regions. Actively transcribed genes are enriched with trimethylation of lysine 4 on histone H3 at their promoters and trimethylation of lysine 36 on histone H3 in their coding regions [32], which are collectively called K4-K36 domains. Taking advantage of this knowledge, Guttman et al. prepared DNA tiling arrays with 2.1 million oligonucleotide probes representing 350 K3-K36 domains and hybridized them with polyadenylated RNA to identify 1,600 mouse lincRNAs [24]. A similar tiling array was used to identify 300 lincRNAs in human cells [23]. Thus, the tiling array approach is highly useful to comprehensively detect any transcripts, including lncRNAs, transcribed from a defined DNA region at a high resolution in an unbiased manner. However, unless the target region is reasonably limited, a potential drawback of the tiling array approach is its high cost. Tiling arrays generally need to be custom-made to meet diverse needs, which further raises the cost and slows down manufacturing the arrays.

## Identification of lncRNAs with RNA-seq

RNA-seq is a powerful tool based on the principles of next-generation sequencing that can be applied to the detection and quantification of lncRNAs. Some advantages of using RNA-seq over a microarrary-based approach are that RNA-seq works on a genome-wide scale at single nucleotide resolution and is not limited to detecting already known sequences. Thus, it can be used to discover previously unknown lncRNAs in an unbiased manner [33]. However, the time and cost related to the downstream analysis of the data generated by RNA-seq is a considerable disadvantage of this approach.

Before beginning RNA-seq, one must decide whether to use total RNA or polyadenylated RNA. The presence of rRNA (around 80-85% of total RNA) and tRNA (15%) [34,35] can drastically reduce the diversity of a cDNA library during amplification of cDNAs. Polyadenylated RNA is frequently used for RNA-seq to avoid this problem. However, given the prevalence of non-polyadenylated lncRNA in the genome (around 40% of total lncRNAs), the disadvantage of losing this fraction is not negligible [36]. One solution to this problem is to use commercially available kits to remove rRNA from total RNA without losing non-polyadenylated RNA.

After sequencing, the generated reads are typically aligned to the UCSC mouse mm10 or human hg19 reference genomes using software programs such as the short-read mappers Bowtie 2 [37] and Burrows-Wheeler Aligner [38], and the splice-junction identifier TopHat [39]. Next, the reads are used to assemble a transcriptome and discover previously unannotated transcripts with programs such as Cufflinks [40], which relies on a reference annotation database, or Scripture, which builds the transcriptome *ab initio*[41]. From here, novel lncRNAs can be identified by excluding protein-coding transcripts and annotated lncRNAs based on the databases of RefSeq, ENCODE, and FANTOM (Functional Annotation of the Mammalian Genome) [42], as well as the two databases of experimentally verified lncRNAs generated by the Mattick lab: lncRNAdb [43] and NRED (Noncoding RNA Expression Database) [44].

Novel lncRNAs often undergo further scrutiny to verify that they are not transcriptional noise and that they indeed do not encode proteins. For instance, if the candidate is located within a K4-K36 domain and enriched with RNA polymerase II binding sites and DNase I hypersensitivity sites (a sign of open chromatin) as detected with the ENCODE data, the candidate is likely to be a product of active transcription [25,26,29]. The protein-coding potential of a candidate lncRNA can be evaluated with the Coding Potential Calculator (CPC) algorithm and other programs [45,46]. However, this is not a straightforward task as detailed in a recent review article [47].

## Conclusions

The recent identification of the genome-wide human lncRNA loci by the ENCODE project is undoubtedly a milestone toward the long-term goal of understanding the functional significance of lncRNAs in many biological phenomena. Applications of microarrays containing these probes will certainly lower the threshold of launching new studies of lncRNAs. However, the use of tiling arrays and RNA-seq will continue to be required to identify splicing variants and tissue-specific lncRNAs. In addition, because of the low conservation of lncRNA sequences across species, the use of these approaches in new species will remain necessary until their ENCODE equivalents become publicly available. Furthermore, we expect that additional technological innovations geared toward studying lncRNAs will continuously emerge to support the rapid development of this fascinating research field.

## Abbreviations

ChIP: Chromatin immunoprecipitation; CLIP: Cross-linking and immunoprecipitation; ENCODE: Encyclopedia of DNA Elements; FANTOM: Functional annotation of the mammalian genome; lincRNA: Large intergenic noncoding RNA; lncRNA: Long noncoding RNA; NRED: Noncoding RNA Expression Database; RefSeq: Reference Sequence; RIP: RNA immunoprecipitation; RNA-seq: RNA sequencing; UV: Ultraviolet.

## Competing interests

The authors declare that they have no competing interests.

## Authors’ contributions

CL and NK wrote and edited the drafts of the paper. Both authors read and approved the final manuscript.
